# The Use of Microfluidic Technology for Cancer Applications and Liquid Biopsy

**DOI:** 10.3390/mi9080397

**Published:** 2018-08-10

**Authors:** Arutha Kulasinghe, Hanjie Wu, Chamindie Punyadeera, Majid Ebrahimi Warkiani

**Affiliations:** 1 The School of Biomedical Sciences, Institute of Health and Biomedical Innovation, Queensland University of Technology, Brisbane, QLD 4059, Australia; Arutha.kulasinghe@qut.edu.au (A.K.); chamindie.punyadeera@qut.edu.au (C.P.); 2 The School of Biomedical Engineering, Faculty of Engineering and Internet Technology, University of Technology Sydney, Ultimo, NSW 2007, Australia; hanjie.wu@student.uts.edu.au; 3 Institute of Molecular Medicine, Sechenov First Moscow State University, Moscow 119991, Russia

**Keywords:** microfluidic, lab on a chip, circulating biomarkers, separation, cancer, liquid biopsy

## Abstract

There is growing awareness for the need of early diagnostic tools to aid in point-of-care testing in cancer. Tumor biopsy remains the conventional means in which to sample a tumor and often presents with challenges and associated risks. Therefore, alternative sources of tumor biomarkers is needed. Liquid biopsy has gained attention due to its non-invasive sampling of tumor tissue and ability to serially assess disease via a simple blood draw over the course of treatment. Among the leading technologies developing liquid biopsy solutions, microfluidics has recently come to the fore. Microfluidic platforms offer cellular separation and analysis platforms that allow for high throughout, high sensitivity and specificity, low sample volumes and reagent costs and precise liquid controlling capabilities. These characteristics make microfluidic technology a promising tool in separating and analyzing circulating tumor biomarkers for diagnosis, prognosis and monitoring. In this review, the characteristics of three kinds of circulating tumor markers will be described in the context of cancer, circulating tumor cells (CTCs), exosomes, and circulating tumor DNA (ctDNA). The review will focus on how the introduction of microfluidic technologies has improved the separation and analysis of these circulating tumor markers.

## 1. Introduction

The rapid development of cancer biomarker technologies is gradually reshaping both the academic and clinical research areas. As we step into the age of personalised medicine, the need for comprehensive cancer biomarkers has dramatically increased [1]. This was evidenced at the recent American Society for Clinical Oncology (ASCO) 2018 and American Association for Cancer Research (AACR) 2018. Cancer related deaths still remain the second leading cause of non-accidental deaths globally [2]. There is an increasing number of technologies being developed for pre-screening, diagnostic, prognostic, therapy assessment, and monitoring of disease. The progression free survival (PFS) and overall survival (OS) in cancer patients has been reported to be dramatically increased if cancers can be detected at an early stage [3].

To date, there are several forms of direct tumor biopsy. According to the tumor site, bone marrow biopsy, endoscopic biopsy, needle biopsies, and skin biopsy will be performed by clinicians in direct assessment of the tumor bulk. In most cases of solid tumors, an image-assisted core needle biopsy is performed, using needles to extract a column of tissue, followed by tissue interrogation by histopathologists [4]. Tissue biopsy remains the gold standard when diagnosing cancer [5]. To determine whether the abnormal tissue is malignant, clinicians have to perform invasive procedures to obtain a small portion of tumor tissue which is later confirmed by histopathology, cytology, and molecular/cytogenetically. It is currently the only way of validating whether the suspicious tissue is cancerous, and can be an invasive procedure for patients to endure [6]. In some cases, it has been reported that the procedure may induce risk of bleeding, inflammation, and even dissemination of cancer cells by providing alternative routes for spreading [7]. Furthermore, repeat tissue biopsy is not always a possibility. Therefore, traditional tumor biopsy only provides a static tumoral snapshot of a specific time point, and does not reflect dynamic changes that occur during cancer treatment [8]. Moreover, the time-to-result procedure takes several days and sometimes weeks to reach clinicians [9]. A study assessing the patient outcomes and the economic implications of utilizes a serum proteomic test to guide the treatment in non-small cell lung cancer. It shows that the blood test resulted in an improved OS rate along with the total lifetime, and the direct medical cost decreased by $135 (U.S. dollars) per patient with test-guided treatment [10]. Tumor heterogeneity is an added challenge with tumor biopsy. Sampling a tumor using a single site biopsy is akin to looking through a keyhole [11,12]. Therefore, the tumor samples obtained by needle biopsy may only represent a small proportion of the whole tumor, which leads to an over estimation of the clonal populations in the tumor bulk. Liquid biopsy may overcome the disadvantages of conventional tumor biopsy methods. Circulating tumor cells (CTCs), circulating tumor DNA (ctDNA) and exosomes which are easily assessable by a simple blood draw, present an attractive alternative to tissue biopsies, and may represent the primary and metastatic tumor sites [13].

As a result of highly sensitive assays and innovative detection platforms, the promise of a liquid biopsy is now closer to reality [14]. Compared to conventional tissue biopsy, a liquid biopsy offers a multi-parameter approach to assess targets for therapy on CTCs, cell-free ctDNA, and exosomes secreted from tumors during the metastatic process [5]. Blood sampling is a non-invasive process and it avoids the complications of traditional tissue biopsies. Liquid biopsy provides an alternative sample type for routine clinical practice when tumor sampling is unavailable, inappropriate, or difficult to obtain. Moreover, it may be possible to assess the dynamic tumoral changes via CTCs and ctDNA over the course of therapy and when treatment resistance becomes a challenge. The analysis of liquid biopsy could be used to provide early cues into alternate therapies before the tumor relapse is detected by conventional methods [13]. To this end, microfluidic technologies have come to the fore in the last few years, where the convenience of such technologies is surpassing that of conventional laboratory bench techniques, such as flow cytometry. Microfluidics provide miniaturized devices that offer fast isolation speeds with high efficiencies and automation, which can be integrated rapidly into multiple workflows [15]. The microfluidic technologies have numerous advantages over conventional methods by reducing the size of equipment, eliminating complex protocols for cell sorting, and allowing for parallelization, which can enable complete lab-on-a-chip devices [16].

Remarkable progress has been achieved in the last decade in the field of microfluidics, leading to applications in single cell analysis and next-generation sequencing technology. With these technological advances, applications have been found for liquid biopsy, which have been reviewed recently [17,18,19], particularly in context of device operation and modalities for CTC sorting. In this review, our intention is to focus more on recent clinical works, and to further highlight the emerging role of microfluidics in order to capture and detect the circulating biomarkers (CTCs, ctDNA, and exosomes), with a focus on the most analytically valid systems. Furthermore, this review provides insight into how microfluidic technologies can be implemented into existing clinical workflows, providing a personalized medicine approach.

## 2. Circulating Biomarkers in Liquid Biopsy. Molecular, Biology, and Morphology Characteristics of CTC, ctDNA, and Exosomes

### 2.1. Circulating Tumor Cells (CTCs)

Circulating tumor cells were first observed by Thomas Ashworth, an Australian physician, who made the incredible finding that cells identical to the tumor itself were found in the blood of a patient with metastatic disease [20].Since this discovery, the field remained in its infancy until about the last 20 years, where there has been an exponential increase in CTC research. This is primarily due to the improvements in rare cell capture from the blood. As of August 2015, there have been 16,688 publications reported on CTCs, 1248 of these were published in 2014, reflecting the advancement of the field over recent years. With the advent of the CellSearch (Menarini-Silicon Biosystems, Bologna, Italy), the first and only Food and Drug Administration (FDA) approved CTC enrichment and enumeration technology came to the clinical setting. In the CellSearch platform, preselected tumor cells for epithelial cell adhesion molecules (EpCAM) and EpCAM-positive CTCs were found to correlate to clinical outcomes. The CTC thresholds were established where five or more CTCs/7.5 mL blood for breast and prostate, and three or more CTCs/7.5 mL blood draw for colorectal cancer were associated with an unfavorable patient prognosis [21]. These findings coined the term ‘liquid-biopsy’, where CTCs could be used to determine the tumorigenic potential non-invasively [22]. Whilst enumeration by CellSearch correlated to the clinical outcomes, numerous limitations were found with the technology. There was a drive to investigate beyond the simple enumeration of CTCs. Moreover, EpCAM was shown to be downregulated in the CTCs during the process of epithelial to mesenchymal transition (EMT), as reported by Yu et al. [23]. The bottle neck in the field was to increase the number of rare CTCs isolated from a blood sample, as there was an increased demand for intact, live CTCs with which to perform a functional analysis. This was overcome by a number of leading research groups by utilising in vivo and ex vivo culture methodologies [24,25,26]. Subsequently, this increase in the critical mass of the CTCs provided the foundation for the testing of therapies outside the patients’ body [25,26].

### 2.2. Monitoring of Disease

**Figure 1:**
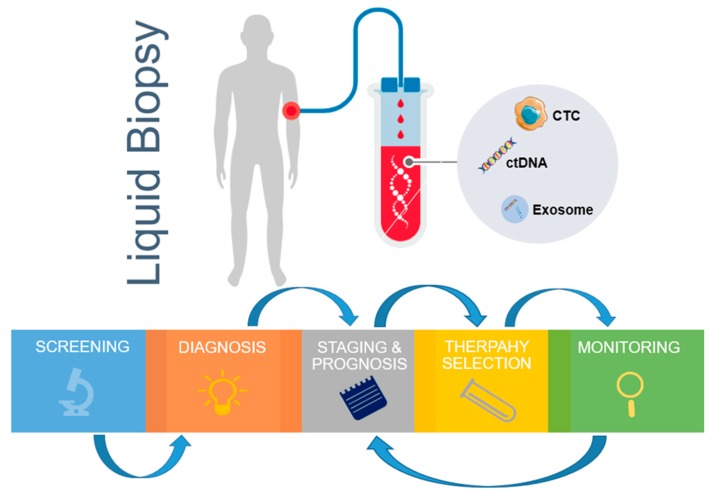
Clinical applications of liquid biopsy from blood circulating markers. The genomics and immunology information derived from liquid biopsy samples can be used for continuous monitoring, from early stage disease screening, assistance diagnosis, personalized therapy selection, to recurrence monitoring. CTC—circulating tumor cells; ctDNA—circulating tumor DNA.

CTCs have been reported to not only be useful prior to treatment, but also over the course of therapy [23]. The ability to non-invasively sample a patient’s tumor by a simple blood draw during treatment provides an attractive solution for disease monitoring (Figure 1). Generally, a decrease in the CTC-numbers (disease burden) has been shown to correspond to better treatment outcomes, and the absence of CTCs post therapy provide a good patient prognosis [21]. Yu et al., 2013, demonstrated in breast cancer that in sequential blood draws from the same patient over the course of therapy, a mesenchymally-shifted CTC population was present post therapy. Therefore, a subset of CTCs with an altered phenotype may remain post treatment. Numerous studies have documented the persistence of stem-like CTCs and mesenchymal CTCs post treatment [27]. Therefore, it becomes critical to profile these residual CTC populations, which may underline the minimal residual disease in patients following therapy.

### 2.3. Epithelial to Mesenchymal Transition (EMT)

Whilst found to occur in embryologic development, gastrulation, and cell plasticity roles, EMT is known to be involved in the process of metastasis. During this process, cancer cells are thought to undergo an epithelial to mesenchymal transition (EMT); down regulate epithelial markers such as EpCAM and E-cadherin [28,29]; lose cell–cell adhesion properties; and become more invasive towards a mesenchymally-shifted phenotype expressing markers such as Vimentin and N-Cadherin. In attaining a partial or complete EMT, CTCs would inherently be missed by epithelial marker selection technologies such as the CellSearch. Therefore, there is an understanding that multi-epitope CTC capture is required to capture the CTC subpopulations at play [30]. Moreover, it has been shown that individual cytokeratin expression is often downregulated during EMT, and a pan-keratin cocktail enables the tracking of partially lost epithelial phenotypes [31,32]. In understanding this biological process, numerous groups around the world moved towards a marker-independent CTC enrichment to capture a greater proportion of CTCs in circulation [33,34,35]. These studies demonstrated that higher numbers of CTCs were captured using label-free methodologies as well as in head to head comparisons with the CellSearch platform, showed higher CTC capture in lung, breast and head and neck cancers [36,37] It is important to note that whilst higher CTC counts are detected by label-free capture, for most cases, it is only the EpCAM-positive CTCs that have demonstrated clinical significance to date.

### 2.4. Biomarkers for Immunotherapy

More recently, immunotherapies have been shown to have durable responses and long-term survival outcomes in a number of tumor types, including melanoma, head and neck, and non-small cell lung cancer [38,39,40]. Immunotherapies such as anti PD-1/PD-L1 have shown highly variable tumoral scoring for eligibility onto PD-1/PD-L1 trials. Studies have shown this to be due to multiple reasons, including tumoral heterogeneity and the dynamic nature of PD-1/PD-L1. Therefore, alternative methods of surveying the PD-1/PD-L1 landscape are needed. Mazel et al., 2015, demonstrated, in a landmark study, the presence of PD-L1 on breast CTCs, and developed a scoring mechanism. In keeping with this, the CellSearch platform has been further developed to include a PD-L1 stain in the blank channel to further characterise EpCAM-positive CTCs. The PD-L1 is concurrently scored against a range of known cell lines. This was further investigated in lung, and head and neck cancers [41]; not only prior to therapy, but over the course of treatment as well [42,43]. The potential for the development a CTC PD-L1 companion diagnostic assay is currently being investigated [44]. Notably, a recent study has shown the presence of PD-1/PD-L1 on CTCs as a result of host-immune interactions [45]. This multitude of recent literature demonstrates the potential immune evasion mechanisms of PD-L1 expressing CTCs.

### 2.5. Exosomes

Exosomes are nano-meter scale, lipid-membrane vesicles that secrete from cells and carry various kinds of molecules such as DNA, RNA, and proteins. Unlike CTCs and ctDNA, which circulate in the body fluid system, the lipid membranes protect biomolecules inside the exosomes. As there are also antigens on the surface of exosomes, widely used separation methods exist that are based on Immunoaffinity. Others methods include size differentiation and ultracentrifugation [46]. Exosomes are a form of extracellular vesicles released by cells, typically with a size of 30–150 nm, and a lipid bilayer. These vesicles contain RNA, DNA, and protein, and are likely to function by removing the excess constituents from cells, and may mediate cell–cell communication [47]. In cancer, exosomes are thought to promote tumor progression [48,49]. Exosomes have been found in numerous body fluids, including blood, urine, amniotic fluid, tears, and breast milk [47]. Studies have demonstrated that in lineage tracing experiments, malignant cancer cell derived exosomes when taken up by benign tumor cells, which induces a conversion to a more malignant phenotype [50]. In metastasis, the exosomes are thought to promote the remodelling of distant, metastasis prone organs [49]. Microfluidics have been employed for the capture of exosomes in liquid biopsy. Numerous technologies exist, such as the ExoSearch chip, which captures blood plasma exosomes combined with multi-marker probing for ovarian cancer [51]. Immunoaffinity can also be utilised in microfluidics devices by manipulating the surfaces with antibodies. Chen et al., 2010 described this using anti-CD63 functionalized surfaces [52]. The high flow rate isolation of exosomes has been shown by Dudani et al., 2015, where polystyrene beads conjugated with anti-human CD63 were embedded in a microfluidic device that used inertial lift forces at a finite Reynolds number in order to position microparticles, and exchanges solutions for rapid purification [53]. To date, the standardisation of exosome isolation protocol is being developed, from ultracentrifugation to microfluidics, and to immuno-affinity capture. Moreover, exosome specific markers are yet to be established and markers indicative of the cell origin are needed.

### 2.6. ctDNA

Circulating tumor DNA is thought to be released from apoptotic tumor cells and necrotic in circulation, and offers valuable information about tumor progression and metastasis. In doing so, ctDNA found in body fluids can carry tumor specific genetic and epigenetic information [54,55]. Most cell-free DNA fragments are 100–200 base pairs generated from cell apoptosis. The concentration of the cell free DNA in cancer patient are generally higher than those in healthy donors, which indicates that the level of cell free DNA may be used for cancer screening [56]. Tumor specific mutations have been identified in cell free DNA samples. Despite this, ctDNA still faces some challenges, such as low ctDNA concentration, the development of allele specific amplification technologies, and high background noise [57]. Recently, there have been landmark studies documenting how ctDNA may be used to phylogenetically profile the subclonal nature of lung cancer relapse and metastasis [53]. Recent data has shown that tumors with a high tumor mutation burden (TMB) have a better response to immunotherapy. This opens up avenues for the assessment of TMB via ctDNA, which is currently being investigated to guide patients for anti PD-1/PD-L1 therapy.

## 3. The Role of Microfluidics in Liquid Biopsy

Microfluidic technologies have come of age in the last 10–15 years with the efficient isolation of tumor cells and cell derived products. Notably, microfluidic platforms offer many advantages over conventional laboratory-based techniques, such as the low volume of samples required and their reagents, high sensitivity, controllable and tunable flow patterns, ease of operation, low costs and the ability to multiplex platforms. Specially, microfluidic devices can be designed to ensure the precision of liquid manipulation, which is not possible with conventional bench-top approaches. This advantage is crucial if low abundance cells and biomolecules are evaluated in a homogeneous fashion. These advances enable the integration of microfluidics into cell biology, genetics, pharmaceutics, and analytic chemistry research. In combination with serial operation units, such as separators, mixers, reactors, and sensors, into one microfluidic chip, a streamlined experimental process could be scaled down to nanolitre-reaction level, and provides researchers with opportunities for analyzing the analytes both spatially and temporally. So far, the majority of microfluidic approach focus on CTC isolation due to the rapid growing interest in cancer diagnosis.

While microfluidic techniques are promising in academic research, the barriers to commercialization cannot be ignored. In terms of the device-related issues, microfluidic platforms that are not integrated need to be connected manually using non-standard laboratory tools. Materials used widely in microfluidic, such as polydimethysiloxane (PDMS), are not suitable for scaling to industrial standards [58]. In addition, the performance of microfluidic platforms are dramatically influenced by air bubbles and small obstructions caused by errors in the operation and the fabrication process. Although some challenges prevent microfluidic technology from achieving its full potential, inspiring discoveries have been shown to revolutionize how we analyze the cells and biomolecules down to single cell level [59].

## 4. Circulating Tumor Cells Separation and Analysis on Microfluidic Chip

### 4.1. Postitive Selection of CTCs

Positive CTC selection is based on the capture of tumor cells using specific surface antigen selection and the exclusion of normal cells. Epithelial cell adhesion molecule (EpCAM) is a widely used marker among all of the immuno-affinity based positive selection approaches. The positive selection of CTCs can be achieved by either coating antibodies onto the reaction channel surface, or by artificially adding antibodies conjugated to micro particles.

#### 4.1.1. Antibody-Coated Microstructure Separation

**Figure 2:**
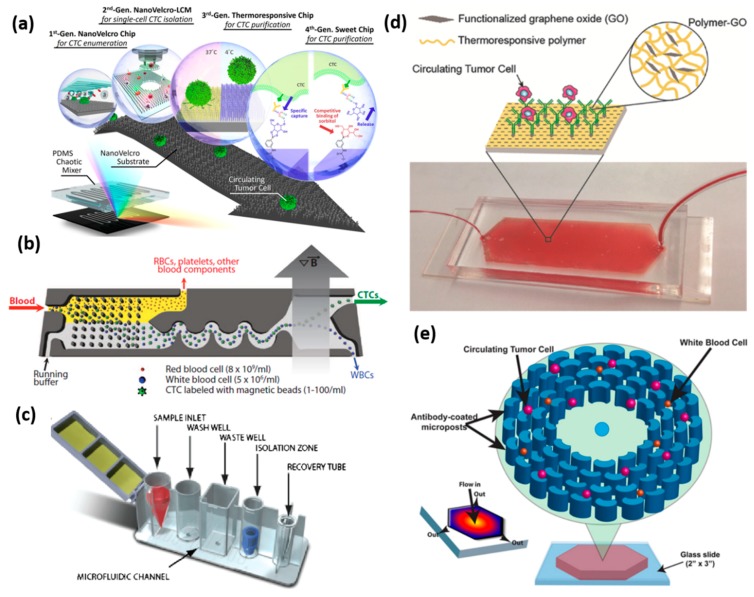
Affinity CTC selection method. (**a**) The graphic illustration of the NanoVelcro CTC chip of four generations. Reprinted with permission from the authors of [63]; (**b**) Schematic of the CTC-iChip. The schematic of the workflow begins from the red blood cell filtration, inertial focusing of CTCs, and white blood cells, followed by the removal of white blood cells under a magnetic field. Reprinted with permission from the authors of [16]; (**c**) The schematic of the microfluidic flow channel of the IsoFlux system. The blood sample mixed with magnetic particles flows through the cartridge, and the circulating tumor cells or other rate cells are enriched and ready for molecular analysis. Reprinted with permission from the authors of [78]; (**d**) Schematic of polymer-graphene oxide (GO) microfluidic device for capturing the CTCs from the patient blood sample. Reprinted with permission from the authors of [61]; (**e**) The illustration of the OncoBean Chip shows that the cancer cells are captured on antibody-modified microposts. The inset heatmap shows the velocity decreasing from the centre to the outer edge. Reprinted with permission from the authors of [62].

EpCAM coated microfluidic channel devices have been used for many years. While the blood samples flow through the microfluidic channel, CTCs are captured against the surface of anti-EpCAM coated microposts. The remaining blood samples are driven away from microfluidic channels. The captured CTC samples could then be stained with fluorescence markers for enumeration purpose, or for undergoing a genomics analysis [60]. However, the application of traditional microfluidic channels is resisted by the small surface area for ligating antibodies, thus resulting in a limited capture capacity. In order to overcome this limitation, various kinds of microfluidic channels have been designed to provide more interactions between the putative CTCs and channel surfaces (Figure 2d,e) [61,62]. Herringbone shape array, micropost array, and nanopillar array could generate a greater surface area in the microfluidic channel, compared with the traditional designs, thus increasing the capture efficiency of the CTCs.

NanoVelcro is a microfluidic chip with nanowires that are manufactured and coated with anti-EpCAM antibody to identify and capture CTCs. While the blood cells flow through the chip, the tumor cells will use immuno-affinity to stick to the nanowires like Velcro. This chip consists of two parts, the bottom substrate is a brush-like silicon nanowire coated with an anti-EpCAM antibody, and a PDMS herringbone structure on the top layer that generates the chaotic flow, which increases the contact chance between CTCs and the antibody substrate at the bottom. After the capture of CTCs, Cytokeratin/CD45/DAPI staining is performed to identify putative CTCs on the chip (Figure 2a) [63].

Capturing the tumor cells is the first part of this platform. In the early stage of NanoVelcro development, researchers used laser capture microdissection to cut out the areas of the CTCs and isolate them from substrate [64]. But this method is time-consuming, and it requires highly specific equipment and experienced operators. Now, researchers have developed thermosensitive NanoVelcro polymer brushes, which are capable of capturing and releasing CTCs at 37 and 4 °C, respectively. The NanoVelcro changes the temperature fluctuations by transforming their physical prosperities, allowing the release of CTCs for downstream molecular assay and cell culture. Furthermore, they fabricated polymer-based nanomaterial on chip surface, the latest NanoVelcro chip is able to purify CTC without damaging RNA transcripts, which benefits the analysis of tumor specific RNA markers (Figure 2a) [65].

#### 4.1.2. In Vivo Antibody-Coated Device Capture

To overcome the restriction of limited blood volume in traditional CTC isolation methods, the GILUPI CellColletor has been designed to collect targeted cells in vivo, by inserting the device through a standard venous cannula into the vein of cancer patients [66]. The device is a 16 cm medical stainless-steel wire with a 2 cm rounded tip that consists of a three-dimensional (3D) anti-EpCAM antibody functioned layer, which comes into contact with circulating blood. Rare CTCs are bound to the device surface by an antigen-anti-EpCAM reaction [67]. While most of the common in vitro detection methods focus on utilizing the small volume of blood efficiently, the total volume of blood passing the GILUPI CellColletor is estimated to be 1.5–3 litres in 30 min, thus increasing the chance of isolating the CTCs [68]. This platform comes into contact with a larger volume of blood compared with the standard 7.5 mL blood volumes, but it is significantly more invasive.

#### 4.1.3. Antibody-Coated Particles Capture Platform

Immunomagnetic bead separation is another popular method for CTC selection. Micro-sized magnetic beads are modified with antibodies that target the surface antigens of CTCs, providing a high detection sensitivity of tumor cells, without the necessary surface modification onto the large surface of a microfluidic channel. In one way, the magnetic beads label the tumor cells by an immune affinity reaction, giving the targeted cells the ability to move under magnetic fields at the next step of magnetic separation. The second way that this method performs is to increase the cell diameter by binding the beads to the cell surface, for the purpose of achieving a higher recovery and purity than the simple filtration separation method [69,70,71]. It total, samples need to be pre-treated with antibodies that are coated with magnetic beads, in order to utilize these immunomagnetic cell separation methods.

CellSearch is still the gold standard for CTC enrichment. It is the first and only clinically validated, FDA-approved blood test for detecting CTCs in cancer patients of metastatic breast, prostate, and colorectal cancer [21]. In brief, ferrofluid nanoparticles coated with antibodies are added to blood samples to enrich for EpCAM-positive CTCs. The CTCs labelled by ferrofluid nanoparticles are then isolated magnetically from the bulk of other cell types in the blood. After CTC isolation, the cells are stained with a pan-cytokeratin, which is a specific marker to identify epithelial CTCs, CD45, which is a specific leukocyte marker to identify any leukocytes that may contaminate the CTC samples and DAPI, a DNA stain to highlight the nuclei of both the CTCs and leukocytes. Before imaging, the cells are put in a magnet cartridge, which will force the cells to form a single focal layer for better imaging results. The fluorescence staining and bright field results of the harvested cells are reviewed, and the CTCs are defined based on their morphology features and staining profiles, which are cytokeratin positive, CD45 negative, and DAPI positive, with a high nuclear to cytoplasmic ratio and a minimum 4 µm × 4 µm diameter [72].

The clinical utility of the CellSearch platform has been validated in many studies [73]. The CTCs number above or below a predetermined threshold can predict the patient prognosis, indicating a favourable or unfavourable prognosis for metastatic breast, prostate, and colorectal cancers patients [21]. For metastasis breast cancer, the patients with a cut-off value of less than five CTCs have a remarkably longer progression free survival [74]. The comprehensive analysis of determining the correlation between the disease progression and the CTC numbers in metastasis prostate and colorectal cancers also demonstrates the clinical utility of CTC, with a cut-off value at five and three CTCs, respectively [75,76].

IsoFlux from Fluxion Biosciences (Alameda, CA, USA) is an automated system that utilizes the microfluidic and immunomagnetic strategy for CTC enrichment. The samples are pushed into the isolation region at a lower speed, to generate a desired reaction time within the isolation region. In the isolation region, a removable, low adherence polymer disk is place below a magnet. A high magnetic field attracts the CTCs labelled with anti-EpCAM coated magnetic beads to reach the polymer disk on the roof. The unbound cells continue flowing through the microfluidic channel to the waste well (Figure 2c) [77,78]. IsoFlux displays higher sensitivity in the identification of CTCs from hepatocellular carcinoma patients, with a detection rate of 4.7% (1/21) by the CellSearch system, and 90.5% (19/21) by the IsoFlux method [79]. The results obtained by both systems are not consistent for the CTC study in hepatocellular carcinoma patients.

### 4.2. Negative Seletion of CTCs

As the understanding of the tumor heterogeneity has grown in recent years, researchers have found out that tumors develop as complex heterogeneous tissue, rather than as one particular clone [80]. Even within the cancer cell lines, the concentration of the surface antigens may vary from cell to cell [81]. Moreover, the utilization of positive selection requires research of the surface markers, which are expressed ubiquitously across all of the CTCs. This makes it impractical to discover unknown CTC subtypes using positive selection.

Negative selection is widely conducted by applying CD45 antibody-immobilized microfluidic channels. For the purpose of increasing the interaction between the leukocytes and the channel surface, rough and unflat surfaces of microfluidic channels are made using strong acid etching or lithography. An example is the CTC-iChip platform. This platform consists of three parts of microfluidic components, namely, deterministic lateral displacement (DLD), inertial focusing, and magnetophoresis to deplete the blood leukocytes. The CTCs from the blood samples of the cancer patients of non-small cell lung cancer, prostate cancer, breast cancer, and melanoma have been successfully isolated through this CTC-iChip platform (Figure 2b) [35]. Unlike other CTC isolation platforms, the CTC-iChip enables whole blood as an input sample by using a micropillar array, which gradually separates the nucleated cells from the red blood cell and platelets by deterministic lateral displacement size-based sorting. After the hydrodynamic sorting, the remaining cells reach a single cell line arrangement under an inertial focusing step. Under a magnetic field, this platform can either enrich the EpCAM positive labelled CTCs or deplete the CD45 positive leukocytes. A combination of inertial focusing and magnetic separation contributes to the precise concentration of CTC or leukocytes into product or waste collection wells, and also allows for positive-labelled CTC enumeration or the negative depletion of leukocytes for antigen-independent CTC enumeration.

### 4.3. Label-Free Separation of CTCs

#### 4.3.1. Size-Based Filtration

**Figure 3:**
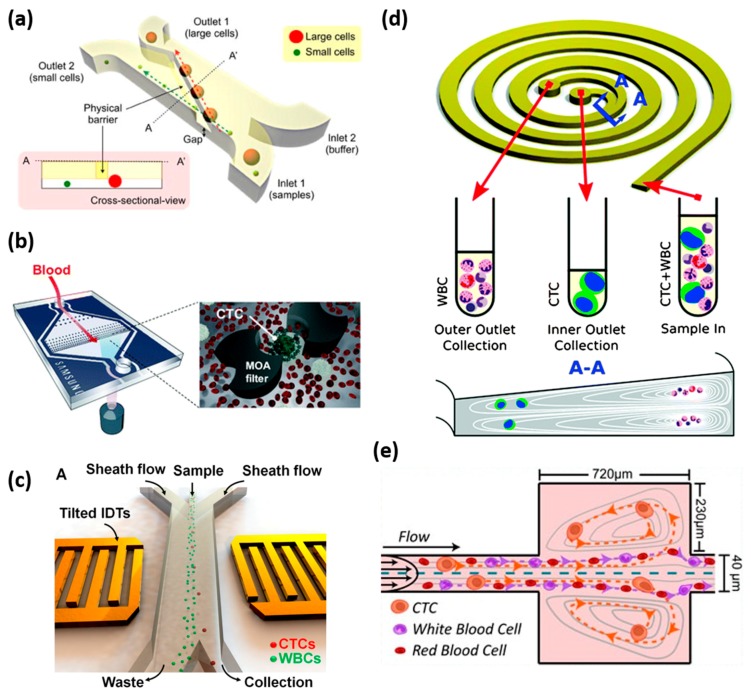
Label-free CTC isolation method. (**a**) Illustration of microfluidic cell sorter. A physical barrier is located, as shown, to separate the cells by size difference. Small cells pass through the gap under the barrier and are collected at outlet 2, while the large cancer cells move along the barrier and are collected at another outlet. Reprinted with permission from the authors of [83]; (**b**) Schematic of the multi-obstacle architecture microfiltration chip for CTC separation. The size gradient filter chip has two filter gaps and the cells are captured between the two filter gaps. Reprinted with permission from the authors of [82]; (**c**) Schematic of acoustic tweezers for isolating circulating tumor cells from a blood sample. These yellow tilted transducers generate soundwaves to move the cells as they pass through the device. The tumor cells are separated from the blood cells based on the difference in size and compressibility. Reprinted with permission from the authors of [91]; (**d**) The principle of CTC enrichment by a spiral channel. The CTCs are focused near the inner wall because of the combination of the inertial lift force and the Dean force, while the white blood cells are focused closer to the outer wall. Reprinted with permission from the authors of [94]; (**e**) Illustration of VTX-1 CTC isolation platform. A blood sample flows through the microchannels and the laminar microscale vortices trap the cells based on their size, shape, and deformability. Reprinted with permission from the authors of [95].

The size based sorting of cancer cells can be performed as a result of the morphologic differences between cancer cells and normal blood cells. CTC capture using microfluidics typically captures CTCs (17–52 µm) that are larger than white blood cells (7–15 µm) and red blood cells (6–8 µm). The CTCs are believed to be inherently larger than the other blood components. Therefore, size-based filtration technologies have been developed for the CTC isolation strategy (Figure 3a,b) [82,83]. VyCap microsieves are fabricated using silicon nitride, with evenly distributed pores of a 5 μm size, in a filter surface of 8 mm × 8 mm, offering 150,000 pores for the rapid blood sample process [84]. For the CTC clusters isolation, the next generation of CTC-chip was designed to provide an easier and more effective isolation effect than the original chip. The two stages of the deterministic lateral displacement strategies that were applied in this chip extract the larger and smaller cluster, maintaining about a 99% recovery rate and over 87% of the cell viabilities [85].

Whilst this works in body fluids such as blood, others, such as urine, can be challenging. For example, malignant urothelial cells that have a cell diameter of 10–436 µm can overlap with exfoliated normal urothelial cells (20–100 µm). Therefore, a combination technologies may need to be used to overcome these challenges, which include a combination of protein expressions [86].

#### 4.3.2. Density-Based Separation

Density-based CTC enrichment is a standardized method for blood component separation. By adding blood above the density gradient reagent and afterwards centrifugation, the blood can be separated into the plasma layer, buffy coat layer containing mononuclear cells, and bottom layer containing red blood cells [87]. This can be used as an initial enrichment step for the CTC isolation and CTC identification through immune-fluorescence staining. However, this CTC enrichment method suffers from a poor retrieval rate and CTC purity. Modifications have been made to improve the performance of this method on CTC isolation [88].

#### 4.3.3. Hydrodynamic-Based Separation

Inertial force, one of the hydrodynamic-based methods, has been shown high throughput separation efficiency, based on particle size variation (Figure 3e) [69]. There are also two types of microchannels for inertial force-based separation, straight channels and curved channels. In the straight channel, the fluid generates lateral forces causing transverse inertial migration of cells. When cells flow though the channels, they need to reach equilibrium at a defined distance between the center of the cell and the microchannel wall under the lateral forces. Larger cells are pushed toward a vortex in the reservoir and trapped inside, while the small size cells will be flushed along the channel, toward the outlet [89]. A spiral microfluidic channel is provided to achieve the separation of CTCs in a curved channel scenario. While the cells flow though the microchannel, the dominant inertial force and rotational flow will force the small size particles to move toward the direction of the outer wall of the channel, and the large size particles migrate toward the inner wall of the channel (Figure 3d) [90]. Thus, this perpendicular Dean-flow can be conducted to separate the cells with a short processing time and high sensitivity.

#### 4.3.4. Acoustic Separation

Peng Li et al. developed an acoustic cell separation device that can manipulate thousands of cells, separating the tumor cells from the blood cells, based on their significant difference in size, compressibility, and other physical properties, by exposing them to sound waves while flowing through the microchannel (Figure 3c) [91]. It can offer a unique approach for researchers in bioengineering projects and clinical diagnosis. Separating the cells with sound offers a gentler alternative to the existing cell sorting techniques, which requires the labelling cells with antibodies or exposing them to stronger mechanical forces that may damage the cells. While there are some white blood cells that have overlapping acoustic properties with cancer cells, a platform, which utilizes the negative acoustic contrast elastomeric particles with CD45-binding whole blood cell (WBCs), was developed to further reduce the WBCs’ background [92]. This platform uses an integration of three modules of microfluidic platforms, which consist of a high-throughput separation, cell spatial organization and cell staining, imaging, and quantification analysis. This platform combines the isolation and evaluation steps towards rare cancer cell sorting and identification. These streamlined functions could be easily extended to various applications on a single cell biology [93].

#### 4.3.5. Magnetophoresis Separation

Magnetophoresis applies the paramagnetic properties of erythrocytes and the diamagnetic properties of other cells, such as leukocyte and tumor cells. In this case, the erythrocytes and biological components could be migrated from the sample under the external magnetic field force. A continuous paramagnetic capture mode of the magnetophoretic microseparator was firstly utilized for the isolation of the suspended breast cancer cells from peripheral blood, based on the native magnetic properties of blood cells, without adding magnetic beads and probes [54]. The remaining nucleated cells, including white blood cells and spiked cancer cells, are detected by a micro-electrical impedance spectroscopy system. It showed that 94.8% of the breast cancer cells from the spiked blood samples were separated and detected.

#### 4.3.6. Dielectrophoresis Separation

When one cell is under the inhomogeneous electric field, it becomes polarized. The polarization interacts with the applied electric field, making each kind of cell perform a unique electrical force, because of the different properties of the cell membrane, cell size, and cell cytoplasmic. When it comes to CTCs, if the electrophoretic of the CTCs are more polarizable than other cells and the medium, then they will migrate towards the electrode side, resulting in separating themselves from the rest of the normal blood cells [96].

## 5. Exosome Separation and Analysis

Exosomes are cell-derived vesicles that carry cell specific protein, lipids, and nucleic acids, that circulate within the body fluids. The cargo that exosomes carry allows for investigation into the tumor origin and potential disease progression. Whilst promising, the translational applications of the exosomes are still a challenge as there are non-standard protocols for the exosome separation and analysis. Despite the conventional exosome separation methods of the ultra-centrifugation and density gradient, the microfluidic methods offer a novel direction for the exosome research area with its less reagent cost, quick processing time, downstream analysis integration ability, and output sample integrity [97]. The size difference and surface biomarkers are two classic factors to isolate the exosomes from the background samples. Thus, the microfluidic platforms for exosome isolation also spits into two streams, namely size-based methods and immuno-affinity-based methods.

### 5.1. Immuno-Affinity Based Separation and Analysis

Similar to the separation method in the CTCs, the immuno-affinity-based exosomes’ isolation and analysis can be achieved by modifying the microchannel channel surface or using antibodies-conjugated microbeads. For the former method, the anti-CD63 antibody was coated onto the surface of the microfluidic channel for capturing exosomes [98]. Then, various antibodies targeting the exosomes’ surface biomarkers are functioned onto the surfaces of different microfluidic devices.

**Figure 4:**
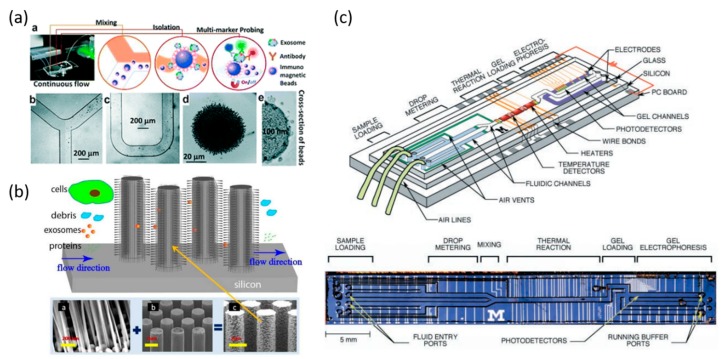
Microfluidic system for exosome and nucleic acid analysis. (**a**) Workflow of microfluidic chip for sample and particle mixing, isolation, and detection of exosomes. Microscope images of immunomagnetic beads for mixing and isolation of exosomes. Reprinted with permission from the authors of [51]; (**b**) A schematic of ciliated micropillar array for exosome separation. While cells are depleted before entering the region, exosomes are highly enriched by trapping within nanowires. Reprinted with permission from the authors of [100]; (**c**) An integrated DNA microfluidic analysis device. (Top) Two liquid samples and electrophoresis gel are presented. (Bottom) Optical micrograph of the device from above. Reprinted with permission from the authors of [111].

However, limited by the surface area of the antibodies immobilization, the efficiency of exosome capture reaches a bottleneck. Therefore, immunomagnetic beads are introduced to overcome this problem. While mixed with exosome samples, the magnetic beads and exosome compound can be considered as a composite in the subsequent magnetic manipulation. The amount of magnetic beads represent the amount of the exosome, thus making the exosome quantitation and analysis possible (Figure 4a) [51]. The immunomagnetic bead method enables an easy sample preparation and rapid exosome enrichment, thus contributing to a higher sensitivity and capture efficiency. To achieve the goal of integrating the exosome integration and chemical lysis, protein immunoprecipitation, and chemiluminescent reactions, a microfluidic chip is developed to capture and analyze the circulating exosome in the plasma [99]. The reported recovery yield of the exosomes using immuno-affinity functionalized CD63 with herringbones groves has been reported to be between 42% and 94% [52]. Other immune-affinity microfluidic technologies include the ExoChip (functionalized CD63 in a mulit-chamber device and nPLEX [functionalized CD24, CD63, and EpCAM] gold surface with nanohole arrays), RInSE (inertial solution exchange for continuous isolation of affinity capture [EpCAM] microbeads), iMER (isolation using magnetic microbeads [EGFR]), ExoSearch (capture of CD9 microbeads), Nano-IMEX (capture of Y-shaped microposts with CD81nanostructured coating), functionalized CD9/CD63 gold surfaces, and µMED (positive and negative enrichment with microbeads of different sizes capturing CD63 particles).

### 5.2. Size-Based Exosomes Separation and Analysis

Isolating exosomes by size-based microfluidic systems mostly relies on the nanoporous structure systems. One classical size-based demonstration is that the nanowire spacing on the micropillars are fabricated to create a high density of physical trapper exosomes [100]. The micropillars shown here work as a scaffold of nanowires, and filter out the large-sized blood cells and other sample components during the exosome separation process. This fabricated microfluidic device selectively traps the exosomes with diameter of 40–100 nm, and filters out proteins, other vesicles, and cell debris at the same time. Moreover, the trapped exosomes remained intact by dissolving into the buffer when compared to the ultracentrifugation methods (Figure 4b) [100]. Novel particle soring techniques are also introduced in the exosome isolation area. An acoustic nanofilter using ultrasound to separate particular particle samples is used, based on their size and density difference. Larger particles experience a stronger radiation force and migrate faster towards the pressure nodes, while the smaller particles remain in the main channel [101]. A novel centrifugal platform utilizes a gentle and efficient size-based exosome separation method without using a syringe pump and other equipment. Combing with table top centrifuge, the extraction of exosome can be achieved in eight minutes, with high separation efficiency of 90% [102].

### 5.3. Other Technologies for Exosome Isolation

Membrane filtration technologies can be used, such as pressure driven filtration using micro/nano-engineered filters [103,104], electrophoresis driven filtration, double filtration (e.g., Exodisc), or nanoporous membranes, which allow for the capture of exosomes [105,106]. Acoustic separation is possible using continuous contact-free nanofilters. For the isolation of urine derived exosomes, the deterministic lateral displacement sorting (NanoDLD) using an array of pillars is possible, and the utilization of inertial focusing using viscoelastic fluid has been also reported [107].

## 6. Circulating Tumor DNA Separation and Analysis

The analysis of ctDNA provides researchers with a further understanding of the basic biological process, prenatal status, and cancer development and progression [108,109]. ctDNA have demonstrated utility in monitoring disease progression over the course of therapy and in the detection of minimal residual disease.

In the conventional methods, electrophoretic and fluorescence signal detection are widely used in DNA analysis. However, inherent limitations, such as the high cost and sample loss, are unavoidable during the multiple sample treatment progress. Therefore, microfluidic devices with integrated functionalities, such as sample preparation, purification, and product analysis, are needed. DNA molecules can be labeled with fluorescent dyes and can be detected with a high signal to noise ratio. Microfluidic platforms for separating nucleic acid have been conducted by many methods, such as silica-based surfaces, functionalized paramagnetic beads, oligonucleotide-modified polymer surfaces, pH-dependent charged surfaces, Al_2_O_3_ membranes, and liquid-phase isolation [110]. However, the processing of reaction products is typically not finished on the microfluidic platform.

One advantage of the microfluidic systems is the ability to combine various techniques into a single integrated and streamlined design (Figure 4c) [111,112]. For example, a highly integrated single molecule DNA analysis platform demonstrated that the polymerase chain reaction (PCR) amplification of a single DNA template is possible, followed by the capillary electrophoretic analysis of the products. The PCR reactions performed in nanoliter chambers can be completed in as fast as 30 s. The fluorescent dye labeled PCR products are then directly injected into the gel-filled capillary channel for electrophoretic analysis [113]. Techniques such as BEAMing (beads, emulsion, amplification, and magnetics) technology incorporate microfluidics to separate the template molecules into individual reaction vessels by separating the sample and PCR reagents into oil droplet emulsions, or by combing the emulsion PCR to generate even sized droplets. BEAMing was one of the first technologies that allowed for the quantitative sensitive interrogation of mutant ctDNA [114]. Digital PCR techniques utilize the limiting dilution of DNA into individual PCR reactions, of which there are two main methods for partitioning individual reactions, namely nanofluidics for well based partitioning and microdroplet partitioning using water-in-oil emulsion. Examples for nanofluidics are the Fluidigm and ThermoFisher OpenArray, and the microdroplet examples are the RainDance and Biorad platforms. These technologies are more precise and demonstrate a linear response to the number of DNA copies, eliminating the need for standard curves to be performed, and enables the use of digital PCR for high resolution determination of the copy number variations (CNVs) [115].

## 7. Microfluidic Techniques in CAR-T Cell Therapy

Immunotherapy has emerged as an important treatment option for cancer. Chimeric antigen receptor (CAR) T cell therapy works using a method of genetically modifying the patient’s own T cells, so that the T cells can recognize and kill the cancer cells without damaging the normal healthy cells. In 2017, there were two CAR-T cell therapies that were approved by the FDA, one for the treatment of children with acute lymphoblastic leukemia, and the other for adults with advanced lymphomas. Moreover, different forms of CAR-T cell therapies are still being developed, including those for solid tumors. However, the current state of engineering the T cells is quite expensive and time-consuming, requiring multiple incubation technologies and highly skilled technicians. To solve these problems, communities from biology and engineering disciplines are working together to develop microfluidic platforms to accelerate this process. Glulia et al. discussed how the 3D microfluidic tumor models mimic the tumor environment, to assess the engineered T cells and improve the therapeutic efficacy [116]. Researchers at Draper are now developing new microfluidic platforms that are able to promote the cell therapies. The first step of this approach is using the acoustics microfluidic platform to enrich the T cells from a patient’s blood sample, achieving rapid and automated T cells separation. After T cells enrichment, the microfluidic chip improves the efficiency of transferring the genetic material into T cells, by reducing the amount of the transferring vector and decreasing the amount of time. This has led to standardization, minimizing the human error and risks associated with cross contamination [117].

## 8. Challenges and Perspectives

Microfluidic technologies will serve an important role in the field of cancer research as well as progressing personalized medicine. In the field of non-invasive liquid biopsy research, CTCs, exosomes, and circulating tumor nucleic acids are used as biomarkers of the minimum residual disease for the monitoring of disease. As a result of different sources and characteristics of these biomarkers, the separation and detection method varies among samples, which leads to inherent strength and limitations, in turn (Table 1). Many studies have validated the potential of microfluidics in biomarkers’ detection, separation, and characterization. Although microfluidic technology shows promise, there are still hurdles to overcome prior to translation into clinic.

CTCs provide the units of metastatic diseases that can be sampled non-invasively by a blood draw. Therefore, the isolation and characterization of CTCs is critical in order to understand the underlying mechanism involved in metastasis. Microfluidic CTC enrichment is categorized into two main streams, label-free techniques and immune-affinity techniques. The ideal microfluidic platform allows for a high CTC recovery rate, high sample throughout volume, and short experimental time.

One appealing advantage of the microfluidic separation techniques is that the high volume and parallel throughout are compatible with the high sample flow rate. Larger sample volumes and higher flow rates make innovative tumor diagnostic methods and therapeutic applications possible [118]. In the case of CTCs, most of the analysis is performed using 7.5 mL of blood. However, recent studies have shown that it is necessary to process larger volumes of blood in order to collect sufficient CTC numbers for reliable enumeration and genomic analysis. Therefore, the microfluidic platforms are ideal to handle and analyze small numbers of cells within large volumes of raw samples. As mentioned above, there are two major types of immuno-affinity-based separation methods, namely positive selection and negative selection. Both are based on either the modification of the microfluidic channels or on the application of antibodies-conjugated to micro-sized magnetic particles. The performance of antibody-modified channels is limited by the interactions between the surface-bound antibodies and the target cells flowing through. The negative selection-based methods allow for a greater capture of the CTC subpopulations, and in so doing, a greater heterogeneity of CTCs than the positive selection methodologies. Whilst there have been developments, there remains a need for improved CTC separation efficiencies for downstream analysis. One method has investigated the surface chemistry of microparticles to provide a short processing time and high CTC recovery rate. Another method utilizes a combination of multiple cell capture technique components, combined into one platform in order to improve the CTC capture performance.

The separation and analysis of the exosome on microfluidic systems may outperform traditional methods, such as ultracentrifugation and some commercially available exosome isolation kits. However, the release of the exosomes of differential sizes and concentrations makes isolating and analysis challenging. The existing exosome microfluidic systems mostly rely on size difference and immuno-affinity techniques. For the former method, the performance of the exosome isolation is greatly affected by the size difference of the secreted exosomes.

In addition, unlike the CTCs, which are of a micro meter size, exosomes are of nanometer size and thereby require more advanced technologies for the device fabrication. Generally, immuno-affinity-based separation methods have a high specificity and sensitivity. However, because of the heterogeneity of the exosomes, there is no common biomarker that can distinguish all of the cancer secreted exosomes from the normal exosomes.

The utility of ctDNA has been shown in identifying minimal residual disease and in the tracking of mutations during therapy. Through the integration of nucleic acid separation, and the analysis on microfluidic systems, these novel platforms show advantages such as low cost, fast processing time, and less contamination, compared with conventional methods. However, there are still some improvements for nucleic acid separation and analysis platforms. Most types of nucleic acid are collected by an extraction process, which will cause damage to the sample integrity. In terms of the circulating nucleic acid separation, there is no standard sample collection and processing protocol, thus limiting the development of reliable and efficient microfluidic platforms.

Overall, the first question comes down to the intrinsic rarity and heterogeneity of the circulating tumor markers. To solve this problem, complexed analysis platforms that combine physical and biological properties of tumor biomarkers need to be developed. Secondly, researchers need to establish the relationship between microfluidic technology and the analysis of the circulating biomarkers. There are a number of liquid biopsy tests that have been approved by FDA so far, such as the breakthrough device from the Foundation Medicine company, approved at 2018; the cobas EGFR mutation test v2, approved for NSCLC in 2017; myRisk, approved for breast cancer in 2014 [119,120,121]. However, these tests mentioned above are based on ctDNA sequencing from plasma samples. Although there are many microfluidic-based liquid biopsy platforms that have shown appealing results, the CellSearch system remains the only FDA-approved CTC platform as of January 2018. To gain more attention from the medical society and to accelerate the FDA approval progress, more validation tests regarding the specificity, sensitivity, and reproducibility of the microfluidic platforms need to be determined for each tumor type, as well as among large populations on current and future microfluidic systems.

## Figures and Tables

**Table 1:** Comparison of three liquid biopsy methods (circulating tumor cells [CTCs], circulating tumor DNA [ctDNA], and exosomes).

Types of Liquid Biopsy Samples	Circulating Tumor Cells	ctDNA	Exosomes
Sample Sources	Peripheral Blood	Plasma or Serum	Plasma or Other Body Fluids
Separation or detection methods	Positive selection by antibody coated microstructure and microparticles	Digital PCR, next generation sequencing, allele-specific PCR	Antibody coated microchannel and microparticles
	Negative selection by antibody coated microstructure and microparticles		Label-free methods: Size based nanowire capture, acoustic nanofilter
	Label-free methods: size-based filtration, density-based separation, hydrodynamic, acoustic, magnetophoresis, de-electrophoretic		
Strength	● High specificity of tumor-derived	● High sensitivity	● Samples available in various body fluids
	● Approved by FDA clinical practice	● Ongoing clinical trials for treatment suggestions	● DNA, RNA, and protein could all be investigated
	● DNA, RNA, and protein could all be investigated		● Functional studies available
	● Functional studies available		
Limitations	● Loss of heterogeneity on non-label free isolation methods	● No RNA or protein could be investigated	● Loss of heterogeneity on non-label free isolation methods
	● Instability of tumor diagnosis at early stage	● Tumor specific mutations are hard to distinguish due to redundant background noise signals	● Absence of specific tumor derived markers
